# Supplementary material for: Multi‐dataset identification of innovative feature genes and molecular mechanisms in keratoconus
Source: J Cell Mol Med. 2024 Sep 19;28(18):e70079. doi: 10.1111/jcmm.70079 (PMC11412914; doi:10.1111/jcmm.70079)
Supplement: Supplementary file 2 — Material S2. [file JCMM-28-e70079-s002.zip › 7.GSEA analysis/6.SNORA74B/KEGG_GLYCOSAMINOGLYCAN_BIOSYNTHESIS_CHONDROITIN_SULFATE.html]

Details for gene set KEGG\_GLYCOSAMINOGLYCAN\_BIOSYNTHESIS\_CHONDROITIN\_SULFATE[GSEA]

|  || Dataset | SNORA74B.group.cls#h\_versus\_l |
| Phenotype | group.cls#h\_versus\_l |
| Upregulated in class | l |
| GeneSet | KEGG\_GLYCOSAMINOGLYCAN\_BIOSYNTHESIS\_CHONDROITIN\_SULFATE |
| Enrichment Score (ES) | -0.5976979 |
| Normalized Enrichment Score (NES) | -1.6054206 |
| Nominal p-value | 0.022140222 |
| FDR q-value | 0.26248536 |
| FWER p-Value | 0.596 |
Table: GSEA Results Summary

  

Fig 1: Enrichment plot: KEGG\_GLYCOSAMINOGLYCAN\_BIOSYNTHESIS\_CHONDROITIN\_SULFATE      
 Profile of the Running ES Score & Positions of GeneSet Members on the Rank Ordered List

  

| SYMBOL | TITLE | RANK IN GENE LIST | RANK METRIC SCORE | RUNNING ES | CORE ENRICHMENT || 1 | CHSY3 | na | 7340 | 0.153 | -0.1691 | No |
| 2 | B3GAT1 | na | 17208 | 0.003 | -0.4526 | No |
| 3 | CHPF | na | 17469 | -0.000 | -0.4601 | No |
| 4 | B3GALT6 | na | 18219 | -0.008 | -0.4793 | No |
| 5 | B3GAT3 | na | 19372 | -0.022 | -0.5065 | No |
| 6 | XYLT2 | na | 19746 | -0.026 | -0.5101 | No |
| 7 | CHSY1 | na | 20647 | -0.036 | -0.5261 | No |
| 8 | CHST12 | na | 21534 | -0.046 | -0.5388 | No |
| 9 | CHST3 | na | 21611 | -0.047 | -0.5279 | No |
| 10 | CHST13 | na | 22581 | -0.059 | -0.5393 | No |
| 11 | DSE | na | 22898 | -0.064 | -0.5307 | No |
| 12 | B4GALT7 | na | 24360 | -0.087 | -0.5487 | No |
| 13 | XYLT1 | na | 25915 | -0.115 | -0.5616 | Yes |
| 14 | B3GAT2 | na | 27170 | -0.143 | -0.5579 | Yes |
| 15 | CHST11 | na | 27526 | -0.152 | -0.5259 | Yes |
| 16 | CHPF2 | na | 28348 | -0.175 | -0.5010 | Yes |
| 17 | CHST7 | na | 31068 | -0.272 | -0.5039 | Yes |
| 18 | CHST15 | na | 31268 | -0.281 | -0.4314 | Yes |
| 19 | CSGALNACT2 | na | 31278 | -0.282 | -0.3533 | Yes |
| 20 | CSGALNACT1 | na | 32691 | -0.367 | -0.2920 | Yes |
| 21 | CHST14 | na | 34088 | -0.554 | -0.1783 | Yes |
| 22 | UST | na | 34517 | -0.708 | 0.0061 | Yes |
Table: GSEA details [plain text format]

  

Fig 2: KEGG\_GLYCOSAMINOGLYCAN\_BIOSYNTHESIS\_CHONDROITIN\_SULFATE      
 Blue-Pink O' Gram in the Space of the Analyzed GeneSet

  

Fig 3: KEGG\_GLYCOSAMINOGLYCAN\_BIOSYNTHESIS\_CHONDROITIN\_SULFATE: Random ES distribution      
 Gene set null distribution of ES for **KEGG\_GLYCOSAMINOGLYCAN\_BIOSYNTHESIS\_CHONDROITIN\_SULFATE**
